# Supplementary material for: New estimates indicate that males are not larger than females in most mammal species
Source: Nat Commun. 2024 Mar 12;15:1872. doi: 10.1038/s41467-024-45739-5 (PMC10933400; doi:10.1038/s41467-024-45739-5)
Supplement: Supplementary file 1 — Supplementary Information [file 41467_2024_45739_MOESM1_ESM.pdf]

New estimates indicate that males are not larger than females in most mammal species

Kaia J. Tombak, Severine B. S. W. Hex, Daniel I. Rubenstein

Supplementary Figures and Tables

Supplementary Fig. 1. Sample size and absolute confidence interval of the intersexual mass difference, relative to mean body mass, for each sex (males in the top panel, females in the bottom panel, N=691 mammal populations in each), with the elbow of the curve marked in red. The elbow was used to determine the minimum sample size for a datapoint to be included in analyses (N=9 males and 9 females).

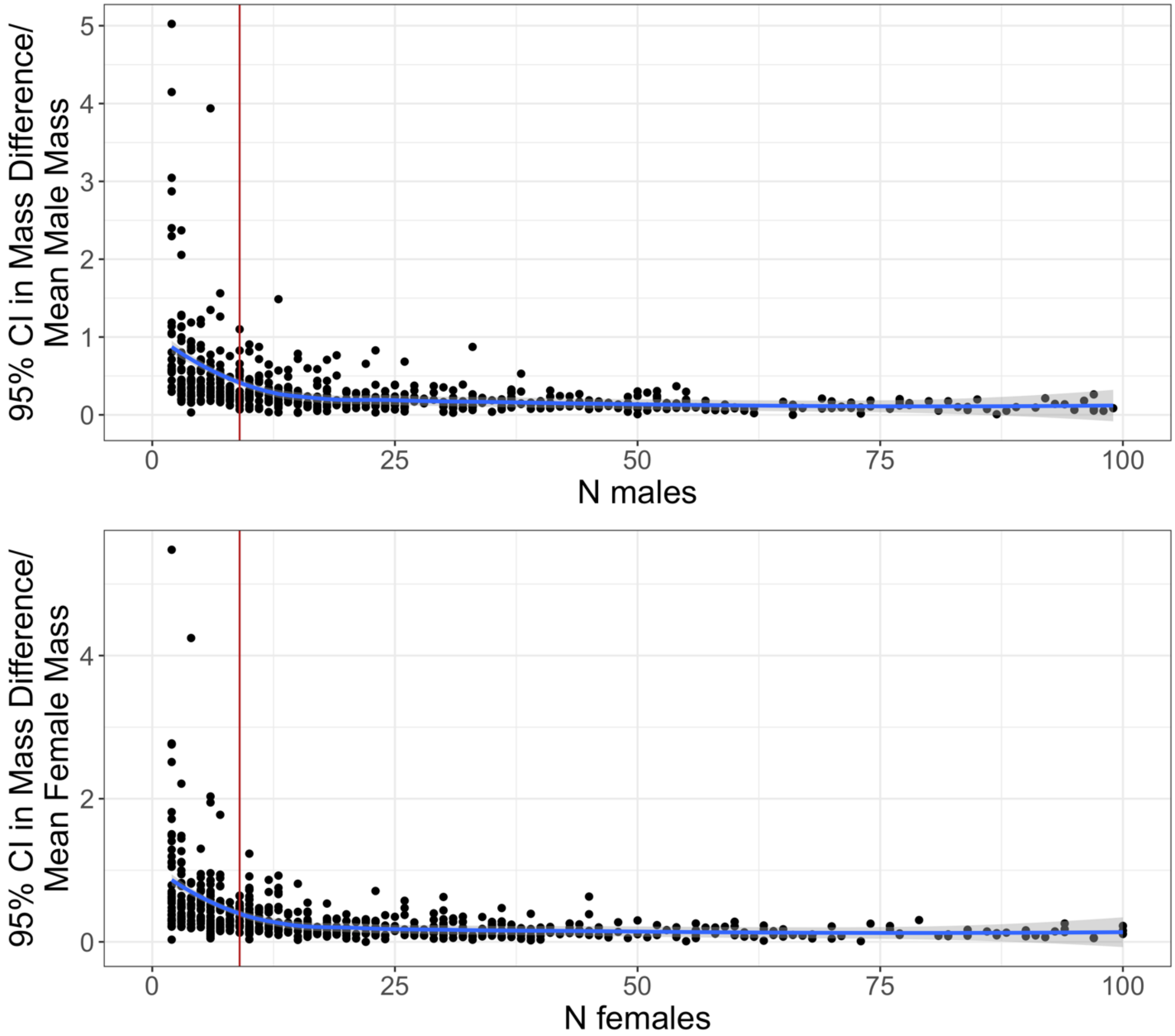

Supplementary Fig. 2. Observed distribution of sexual size dimorphism across mammalian families in each order (only families with at least ten species are shown, in addition to the Pilosa families sampled). Filled bars show the number of species in each category in our dataset, and floating pink bars demarcate the 5% representation goal (i.e., the maximum number of species included in each rate estimation). Please see Supplementary Table 1 for the sample sizes for each family sampled.

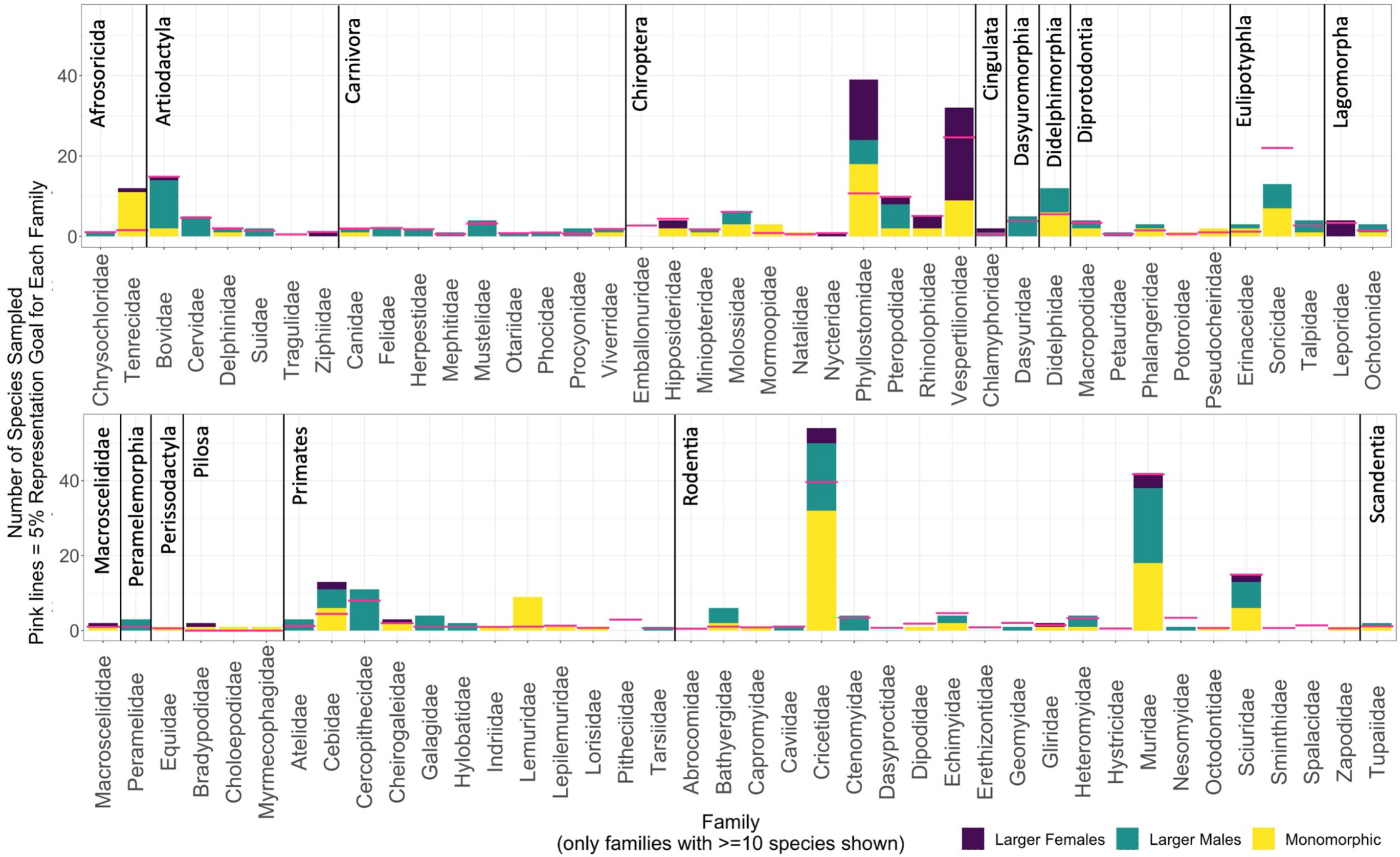

Supplementary Table 1. Summary of sample sizes achieved for each order (in order of species richness) and family, the estimated rates of dimorphism & monomorphism in each order (percentages in bold in colored columns, representing rates calibrated by species richness in each family), and the distribution of dimorphism in the whole dataset by family (numbers in colored columns, representing the number of species in the dataset in each category). We excluded orders and families with <10 species, with the exception of Pilosa, an order of 10 species in which no family had more than 4 species, and a few cases where data from families with <10 species were included to help achieve 5% representation for the order. Under Primates, Cebidae includes Aotidae and Callitrichidae, following Burgin et al.[1].

ORDER<br>FAMILY	DESCRIPTION	# SPECIES SAMPLED/ # EXTANT SPECIES (% REPRESENTED)	LARGER FEMALES<br>EST. % OF ORDER<br># SPP. IN FAMILY	MONOMORPHIC<br>EST. % OF ORDER<br># SPP. IN FAMILY	LARGER MALES<br>EST. % OF ORDER<br>& # SPP. IN FAMILY
**Rodentia**	**Rats, mice, & allies**	**139/2552 (5.4%)**	**8.2%**	**47.6%**	**44.2%**
Abrocomidae	Chinchilla rats	0/10 (0%)	-	-	-
Bathyergidae	African mole rats	6/21 (28.6%)	0	2	4
Capromyidae	Hutias	1/17 (5.9%)	0	1	0
Castoridae	Beavers	1/2 (50%)	0	1	0
Caviidae	Cavies & maras	1/21 (4.8%)	0	0	1
Cricetidae	Hamsters, voles, lemmings, muskrats, New World mice, & rats	54/792 (6.8%)	4	32	18
Ctenomyidae	Tuco-tucos	4/69 (5.8%)	0	0	4
Dasyproctidae	Agoutis & acouchis	0/15 (0%)	-	-	-
Dipodidae	Jerboas	1/37 (2.7%)	0	1	0
Echimyidae	Shiny rats	4/93 (4.3%)	0	2	2
Erethizontidae	New World porcupines	0/17 (0%)	-	-	-
Geomyidae	Gophers	1/41 (2.4%)	0	0	1
Gliridae	Dormice	2/29 (6.9%)	1	1	0
Heteromyidae	Kangaroo rats, kangaroo mice, pocket mice, & spiny pocket mice	4/66 (6.1%)	0	1	3
Muridae	Mice, rats, & gerbils	42/834 (5%)	4	18	20

Nesomyidae	Pouched rats, swamp mice, & allies	1/68 (1.5%)	0	0	1
Octodontidae	Rock rats, coruros, degus, mountain degus, & viscacha rats	1/14 (7.1%)	0	1	0
Sciuridae	Squirrels	15/298 (5%)	2	6	7
Sminthidae	Birch mice	0/14 (0%)	-	-	-
Spalacidae	Blind mole-rats, bamboo rats, mole-rats, & zokors	0/28 (0%)	-	-	-
Zapodidae	Jumping mice	1/12 (8.3%)	0	1	0
**Chiroptera**	**Bats**	**104/1386 (7.5%)**	**45.7%**	**36.6%**	**17.7%**
Emballonuridae	Sac-winged bats	0/54 (0%)	-	-	-
Hipposideridae	Old World leaf-nosed bats	4/88 (4.5%)	2	2	0
Miniopteridae	Bent-winged & long-winged bats	2/35 (5.7%)	0	1	1
Molossidae	Free-tailed bats	6/122 (4.9%)	0	3	3
Mormoopidae	Mustached, ghost-faced, & naked-backed bats	3/17 (17.6%)	0	3	0
Natalidae	Funnel-eared bats	1/11 (9.1%)	0	1	0
Noctilionidae	Bulldog bats	1/2 (50%)	0	0	1
Nycteridae	Slit-faced bats	1/16 (6.3%)	1	0	0
Phyllostomidae	New World leaf-nosed bats	39/214 (18.2%)	15	18	6
Pteropodidae	Old World fruit bats, flying foxes	10/197 (5.1%)	2	2	6
Rhinolophidae	Horseshoe bats	5/102 (4.9%)	3	2	0
Vespertilionidae	Vesper bats	32/493 (6.5%)	23	9	0
**Artiodactyla**	**Even-toed ungulates**	**30/551 (5.4%)**	**8.3%**	**12.5%**	**79.2%**
Bovidae	Buffalo, antelopes, goat-antelopes, & allies	15/297 (5.1%)	1	2	12
Cervidae	True deer & allies	5/93 (5.4%)	0	0	5
Delphinidae	Dolphins	2/40 (5%)	0	1	1
Phocoenidae	Porpoises	2/7 (28.6%)	1	0	1
Pontoporiidae	The La Plata dolphin	1/1 (100%)	0	1	0
Suidae	Pigs	2/28 (7.1%)	0	0	2

Tayassuidae	Peccaries	2/5 (40%)	0	2	0
Tragulidae	Chevrotains	0/10 (0%)	-	-	-
Ziphiidae	Beaked whales	1/22 (4.5%)	1	0	0
**Eulipotyphla**	**Hedgehogs, moles, shrews, & allies**	**20/527 (3.8%)**	**0%**	**49.6%**	**50.4%**
Erinaceidae	Hedgehogs & moonrats	3/24 (12.5%)	0	2	1
Soricidae	Shrews	13/440 (3%)	0	7	6
Talpidae	Moles	4 /54 (7.4%)	0	1	3
**Primates**	**Lemurs, monkeys, apes, & allies**	**50/518 (9.7%)**	**5.7%**	**32.7%**	**61.6%**
Atelidae	Howler, spider, woolly, & woolly spider monkeys	3/23 (13%)	0	0	3
Cebidae	Capuchins & squirrel monkeys, combined here with night monkeys (Aotidae) & marmosets, tamarins, & lion tamarins (Callitrichidae)	13/89 (14.6%)	2	6	5
Cercophithecidae	Old World monkeys	11/160 (6.9%)	0	0	11
Cheirogaleidae	Dwarf & mouse lemurs	3/40 (7.5%)	1	2	0
Galagidae	Bush babies	4/20 (20%)	0	0	4
Hominidae	Great apes	1/7 (14.3%)	0	0	1
Hylobatidae	Gibbons	2/20 (10%)	0	0	2
Indriidae	Wooly lemurs & sifakas	1/19 (5.3%)	0	1	0
Lemuridae	True lemurs, wooly lemurs, & allies	9/21 (4.3%)	0	9	0
Lepilemuridae	Sportive lemurs	1/26 (3.8%)	0	1	0
Lorisidae	Lorises, pottos, & angwantibos	1/15 (6.7%)	0	1	0
Pitheciidae	Titis, sakis, & uakaris	0/58 (0%)	-	-	-
Tarsiidae	Tarsiers	1/13 (7.7%)	0	0	1
**Carnivora**	**Cat-like & dog-like carnivores**	**20/305 (6.6%)**	**0%**	**13.3%**	**86.7%**
Canidae	Dogs, foxes, & allies	2/39 (5.1%)	0	1	1
Eupleridae	Malagasy carnivores	3/8 (37.5%)	0	1	2

Felidae	Cats	2/42 (4.8%)	0	0	2
Herpestidae	Mongooses	2/36 (5.6%)	0	0	2
Mephitidae	Skunks	1/12 (8.3%)	0	0	1
Mustelidae	Weasels, otters, badgers & allies	4/64 (6.3%)	0	0	4
Otariidae	Eared seals	1/16 (6.3%)	0	0	1
Phocidae	Earless seals	1/19 (5.3%)	0	0	1
Procyonidae	Racoons, ringtails, coatis & allies	2/14 (14.3%)	0	0	2
Viverridae	Civets, genets, & allies	2/37 (5.4%)	0	1	1
**Diprotodontia**	**Kangaroos, koalas, & allies**	**14/155 (9%)**	**0%**	**60.3%**	**39.7%**
Burramyidae	Pygmy possums	1/5 (20%)	0	1	0
Macropodidae	Kangaroos, wallabies, quokkas & allies	4/67 (6%)	0	2	2
Petauridae	Trioks & gliders	1/12 (8.3%)	0	0	1
Phalangeridae	Cuscus & brushtail possums	3/30 (10%)	0	2	1
Phascolarctidae	Koalas	1/1 (100%)	0	0	1
Potoroidae	Bettongs, potoroos, & rat-kangaroos	1/12 (8.3%)	0	1	0
Pseudocheiridae	Ringtail possums & allies	2/20 (10%)	0	2	0
Vombatidae	Wombats	1/3 (33.3%)	0	0	1
**Didelphimorphia**	**Opossums**	**12/111 (10.8%)**	**0%**	**50.2%**	**49.8%**
Didelphidae	Opossums	12/111 (10.8%)	0	6	6
**Lagomorpha**	**Rabbits, pikas, & hares**	**7/98 (7.1%)**	**60.0%**	**13.5%**	**26.5%**
Leporidae	Rabbits & hares	4/67 (6%)	4	0	0
Ochotonidae	Pikas	3/30 (10%)	0	1	2
**Dasyuromorphia**	**Carnivorous Australian marsupials**	**5/78 (6.4%)**	**0%**	**0%**	**100%**
Dasyuridae	Carnivorous Australian marsupials	5/76 (6.6%)	0	0	5
**Afrosoricida**	**Tenrecs, otter shrews, & golden moles**	**13/55 (23.6%)**	**5.5%**	**61.2%**	**33.3%**

Chrysochloridae	Golden moles	1/21 (4.8%)	0	0	1
Tenrecidae	Tenrecs	12/31 (38.7%)	1	11	0
**Macroscelidea**	**Elephant shrews**	**2/30 (6.7%)**	**52.0%**	**48.0%**	**0%**
Macroscelididae	Elephant shrews	2/30 (6.7%)	1	1	0
**Scandentia**	**Treeshrews**	**2/24 (8.3%)**	**0%**	**47.7%**	**52.3%**
Tupaiidae	Treeshrews	2/23 (8.7%)	0	1	1
**Peramelemorphia**	**Bandicoots & bilbies**	**3/23 (13%)**	**0%**	**0%**	**100%**
Peramelidae	Bandicoots	3/20 (15%)	0	0	3
**Perissodactyla**	**Odd-toed ungulates**	**1/21 (0%)**	**0%**	**100%***	**0%**
Equidae	Horses, zebras, & asses	1/12 (0%)	0	1	0
Rhinocerotidae	Rhinoceroses	0/5 (0%)	-	-	-
Tapiridae	Tapirs	0/4 (0%)	-	-	-
**Cingulata**	**Armadillos**	**3/20 (15%)**	**52.7%**	**0%**	**47.3%**
Chlamyphoridae	Armadillos	2/13 (15%)	1	0	1
Dasypodidae	Armadillos	1/7 (14.3%)	0	1	0
**Pilosa**	**Anteaters & sloths**	**4/10 (40%)**	**21.9%**	**78.1%**	**0%**
Bradypodidae	Three-toed sloths	2/4 (50%)	1	1	0
Choloepodidae	Two-toed sloths	1/1 (100%)	0	1	0
Cylcopedidae	Silky anteater	0/1 (0%)	-	-	-
Myrmecophagidae	Anteaters	1/3 (33%)	0	1	0

*While we found only one study on perissodactyls that met our criteria for inclusion in analyses (on plains zebras, *Equus quagga*, which were found to be monomorphic[2]), several studies reported data or analyses indicating monomorphism in other perissodactyls (Przewalski's horses, *Equus ferus*[3], Indian rhinoceros, *Rhinoceros unicornis*[4], black rhinoceros, *Diceros bicornis*[5], and South American tapirs, *Tapirus terrestris*[6]).

References

1. Burgin, C. J., Colella, J. P., Kahn, P. L. & Upham, N. S. How many species of mammals are there? *J. Mammal.* **99**, 1–14 (2018).
2. Smuts, G. L. Pre- and postnatal growth phenomena of Burchell's zebra *Equus burchelli antiquorum*. *Koedoe* **18**, 69–102 (1975).
3. Allen, J. L. Immobilization of Mongolian wild horses (*Equus przewalskii przewalskii*) with carfentanil and antagonism with naltrexone. *J. Zoo Wildl. Med.* **23**, 422–425 (1992).
4. Harthoorn, A. M. Capture of the white (square-lipped) rhinoceros *Ceratotherium simum simum* (Burchell) with the use of drug immobilization technique. *Can. J. Comp. Med. Vet. Sci.* **26**, 203–8 (1962).
5. Berger, J. & Cunningham, C. Predation, sensitivity, and sex: why female black rhinoceroses outlive males. *Behav. Ecol.* **6**, 57–64 (1995).
6. Gonçalves, F. *et al.* Atlantic Mammal Traits: a data set of morphological traits of mammals in the Atlantic Forest of South America. *Ecology* **99**, 498 (2018).
