# Supplementary material for: New estimates indicate that males are not larger than females in most mammal species
Source: Nat Commun. 2024 Mar 12;15:1872. doi: 10.1038/s41467-024-45739-5 (PMC10933400; doi:10.1038/s41467-024-45739-5)
Supplement: Supplementary file 3 — Reporting Summary [file 41467_2024_45739_MOESM3_ESM.pdf]

nature portfolio

Corresponding author(s): Kaia Tombak

Last updated by author(s): Jan 11, 2024

Reporting Summary

Nature Portfolio wishes to improve the reproducibility of the work that we publish. This form provides structure for consistency and transparency in reporting. For further information on Nature Portfolio policies, see our Editorial Policies and the Editorial Policy Checklist.

Statistics

For all statistical analyses, confirm that the following items are present in the figure legend, table legend, main text, or Methods section.

n/a	Confirmed	
☐	☒	The exact sample size (*n*) for each experimental group/condition, given as a discrete number and unit of measurement
☐	☒	A statement on whether measurements were taken from distinct samples or whether the same sample was measured repeatedly
☐	☒	The statistical test(s) used AND whether they are one- or two-sided<br>*Only common tests should be described solely by name; describe more complex techniques in the Methods section.*
☐	☒	A description of all covariates tested
☐	☒	A description of any assumptions or corrections, such as tests of normality and adjustment for multiple comparisons
☐	☒	A full description of the statistical parameters including central tendency (e.g. means) or other basic estimates (e.g. regression coefficient) AND variation (e.g. standard deviation) or associated estimates of uncertainty (e.g. confidence intervals)
☐	☒	For null hypothesis testing, the test statistic (e.g. *F*, *t*, *r*) with confidence intervals, effect sizes, degrees of freedom and *P* value noted<br>*Give P values as exact values whenever suitable.*
☒	☐	For Bayesian analysis, information on the choice of priors and Markov chain Monte Carlo settings
☒	☐	For hierarchical and complex designs, identification of the appropriate level for tests and full reporting of outcomes
☒	☐	Estimates of effect sizes (e.g. Cohen's *d*, Pearson's *r*), indicating how they were calculated

Our web collection on statistics for biologists contains articles on many of the points above.

Software and code

Policy information about availability of computer code

Data collection	All data were collected from the literature, using Google Scholar as a search tool.
Data analysis	All data analyses were performed using R Studio (version 4.3.2), and we used standard packages to organize the data (dplyr, tidyr), to visualize the data (ggplot2, ggExtra, see), and one specialized package (KneeArrower), all specified in the manuscript, to find the elbow in the curve between sample size and confidence interval in order to determine our minimum sample size threshold for inclusion. The R code for our data analyses are also publicly available on Zenodo.

For manuscripts utilizing custom algorithms or software that are central to the research but not yet described in published literature, software must be made available to editors and reviewers. We strongly encourage code deposition in a community repository (e.g. GitHub). See the Nature Portfolio guidelines for submitting code & software for further information.

Data

Policy information about availability of data

All manuscripts must include a data availability statement. This statement should provide the following information, where applicable:

- Accession codes, unique identifiers, or web links for publicly available datasets
- A description of any restrictions on data availability
- For clinical datasets or third party data, please ensure that the statement adheres to our policy

The data generated in this study, as well as the associated list of data sources, are publicly available on Dryad at: https://doi.org/10.5061/dryad.280gb5mx085. A

summary table and bar chart of the results for each order and family may be found in the supplementary materials (Supplementary Table 1, Supplementary Fig. 2). Any correspondence related to the manuscript should be addressed to KJT at ktombak@alumni.princeton.edu.

Research involving human participants, their data, or biological material

Policy information about studies with human participants or human data. See also policy information about sex, gender (identity/presentation), and sexual orientation and race, ethnicity and racism.

Reporting on sex and gender	No data on humans were used in this study.
Reporting on race, ethnicity, or other socially relevant groupings	No data on humans were used in this study.
Population characteristics	No data on humans were used in this study.
Recruitment	No data on humans were used in this study.
Ethics oversight	No data on humans were used in this study.

Note that full information on the approval of the study protocol must also be provided in the manuscript.

Field-specific reporting

Please select the one below that is the best fit for your research. If you are not sure, read the appropriate sections before making your selection.

☐ Life sciences ☐ Behavioural & social sciences ☒ Ecological, evolutionary & environmental sciences

For a reference copy of the document with all sections, see nature.com/documents/nr-reporting-summary-flat.pdf

Ecological, evolutionary & environmental sciences study design

All studies must disclose on these points even when the disclosure is negative.

Study description	This study aimed to estimate the rate of sexual size dimorphism in body mass in mammals, sampling each mammalian order and family according to its species richness and determining sexual size dimorphism for each species statistically. We used data collected from the literature on sex-segregated means and variances in body mass, filtered the full dataset for a minimum sample size of 9 males and 9 females (the threshold beyond which 95% confidence intervals were drastically less inflated by low sample size), and then statistically sampled all data collected according to the species richness in each family to mitigate any overrepresentation by better-studied taxa. Our full dataset, before filtering, included data from 691 populations from 630 unique species, and after sample size filtering and removal of species duplicates (keeping only data with the highest sample size for any species for which more than one population was sampled), we ran our analyses on a dataset of 429 species.
Research sample	Our research sample included any mammalian species for which sex-segregated body mass data derived from wild, adult individuals were available in the literature. The data sources included 8 pre-existing datasets that reported both means and variances for each sex for a large number of species, as well as 266 primary sources reporting such data on various mammalian taxa.
Sampling strategy	Using Google Scholar, we initially sampled the literature broadly to capture any data available in pre-existing datasets. We then continued our search through primary sources, allocating our sampling effort to each mammalian taxon according to its species richness. During the data collection process, a minimum sample size of 2 males and 2 females was used because standard deviation cannot be calculated from 1 sample. However, after data were collected, we statistically determined a threshold for minimum sample size for analyses (using the elbow of the curve between 95% CIs and sample size to determine the point at which confindence interval inflation was drastically reduced by low sample size). Based on this analysis, we used only data with a minimum sample size of 9 males and 9 females for our analyses on rates of sexual size dimorphism in mammals.
Data collection	KJT and SBSWH collected data from the literature. We began by collecting data from pre-existing datasets where sex-segregated body mass means and variances were reported for many species at a time. From these data, we estimated that a feasible goal for the proportion of representation from each mammalian order and family for our study would be about 5%. Using this goal, we searched the literature in a more targeted way, searching the primary literature in an attempt to reach 5% of the number of species from each mammalian family in which there are at least 10 species (because 10 is the minimum number for which 5% rounds up to 1). We reached this goal for 85% of mammalian families, and for all orders except for Eulipotyphla, for which enough data are not currently available in the literature to cover 5% of the species in the order.
Timing and spatial scale	Data were taken from any wild mammal population anywhere in the world, collected at any time in the past. Our literature search began in June 2021 an ended in December 2023.
Data exclusions	Exclusion criteria were pre-established and based on avoiding confounds to naturally-occurring sexual size dimorphism or monomorphism in body mass. We did not use data derived from captive animals, food-provisioned populations, sexually immature individuals, or pregnant individuals (with a few exceptions, where very early pregnancies or very few pregnant individuals were

	included in the mix and unlikely to affect the results). Data from museum specimens were generally not used except where body masses were verified from field data and collected from individuals in the same population.
Reproducibility	All raw data and R code (including detailed justifications and descriptions of each step) used in the analyses are provided and fully reproducible. We tested the code on multiple computers and the results were the same.
Randomization	We are not reporting on an experiment and so there are no treatment groups in our study. Species were grouped into categories of sexual size dimorphism or monomorphism by statistically testing for differences in body mass between the sexes.
Blinding	Blinding was not applicable to our study as we used all relevant data that we could find in the literature in our initial analyses (i.e., there was no 'experiment' per se, in which observers could be blind to the variables). However, we did not know a priori whether any given species for which we were collecting data would be considered sexually dimorphic, as this was tested using 95% CIs calculated after the data were gathered and after data were filtered by sample size according to our preliminary analyses on where the 'elbow' of the curve between 95% CIs and sample size lay using the full dataset. In that sense, all observers were blind to how the inclusion of any given species would affect the results as data were gathered from the literature.

Did the study involve field work? ☐ Yes ☒ No

Reporting for specific materials, systems and methods

We require information from authors about some types of materials, experimental systems and methods used in many studies. Here, indicate whether each material, system or method listed is relevant to your study. If you are not sure if a list item applies to your research, read the appropriate section before selecting a response.

Materials & experimental systems

n/a	Involved in the study
☒	☐ Antibodies
☒	☐ Eukaryotic cell lines
☒	☐ Palaeontology and archaeology
☐	☒ Animals and other organisms
☒	☐ Clinical data
☒	☐ Dual use research of concern
☒	☐ Plants

Methods

n/a	Involved in the study
☒	☐ ChIP-seq
☒	☐ Flow cytometry
☒	☐ MRI-based neuroimaging

Animals and other research organisms

Policy information about studies involving animals; ARRIVE guidelines recommended for reporting animal research, and Sex and Gender in Research

Laboratory animals	*For laboratory animals, report species, strain and age OR state that the study did not involve laboratory animals.*
Wild animals	While no new data were collected for this study, we collected data on wild animal populations reported in the literature.
Reporting on sex	Our study specifically collected sex-segregated data from wild animal populations in order to test long-standing assumptions about sexual size dimorphism in mammals, and all of our analyses are based on sex. We only used data that were separated by sex (means, standard deviations, and sample sizes) and trusted the authors of the studies in question with respect to sex assignment (most studies from which data were collected were conducted by specialists on the taxon in question). We have provided our full, sex-segregated dataset, which is publicly available on Dryad.
Field-collected samples	*For laboratory work with field-collected samples, describe all relevant parameters such as housing, maintenance, temperature, photoperiod and end-of-experiment protocol OR state that the study did not involve samples collected from the field.*
Ethics oversight	*Identify the organization(s) that approved or provided guidance on the study protocol, OR state that no ethical approval or guidance was required and explain why not.*

Note that full information on the approval of the study protocol must also be provided in the manuscript.

Plants

Seed stocks	*Report on the source of all seed stocks or other plant material used. If applicable, state the seed stock centre and catalogue number. If plant specimens were collected from the field, describe the collection location, date and sampling procedures.*
Novel plant genotypes	*Describe the methods by which all novel plant genotypes were produced. This includes those generated by transgenic approaches, gene editing, chemical/radiation-based mutagenesis and hybridization. For transgenic lines, describe the transformation method, the number of independent lines analyzed and the generation upon which experiments were performed. For gene-edited lines, describe the editor used, the endogenous sequence targeted for editing, the targeting guide RNA sequence (if applicable) and how the editor was applied.*
Authentication	*Describe any authentication procedures for each seed stock used or novel genotype generated. Describe any experiments used to assess the effect of a mutation and, where applicable, how potential secondary effects (e.g. second site T-DNA insertions, mosiacism, off-target gene editing) were examined.*
